# Efficacy and safety of intravenous mesenchymal stem cells for ischemic stroke patients, a systematic review and meta-analysis

**DOI:** 10.3389/fstro.2023.1339331

**Published:** 2024-01-17

**Authors:** Maged Elsayed Hassanein, Jaber Fayad, Jilan Ali Shabana, Esraa M. AlEdani, Mahmoud Tarek Hefnawy, Hazem S. Ghaith, Ahmed Negida

**Affiliations:** ^1^Faculty of Medicine, Zagazig University, Zagazig, Egypt; ^2^Klinik für Neurologie und Neurophysiologie, Katholisches Klinikum Essen, Philippusstift, Essen, Germany; ^3^Faculty of Medicine, Basra University, Basra, Iraq; ^4^Faculty of Medicine, Al-Azhar University, Cairo, Egypt

**Keywords:** humans, ischemic stroke, cerebral infarction, mesenchymal stem cells, stroke

## Abstract

**Background:**

Clinical trials have evaluated the efficacy of intravenous mesenchymal stem cells (MSCs) for acute and subacute ischemic stroke. Therefore, we conducted this meta-analysis to investigate the efficacy and safety of intravenous MSC treatments compared to placebo for acute and subacute ischemic stroke patients.

**Methods:**

We searched PubMed, SCOPUS, Web of Science, and Cochrane CENTRAL for randomized controlled trials evaluating any clinical trials of intravenous MSCs for acute and subacute ischemic stroke patients. The efficacy outcomes of this study were the rates of improvement in National Institutes of Health Stroke Scale (NIHSS) scores, good scores on the modified Rankin Scale (mRS), and Barthel Index (BI) scores, while the safety outcomes were the rates of mortality and stroke recurrence. We compared intravenous MSC and placebo treatments on a fixed-effect meta-analysis model in R software.

**Results:**

Four randomized controlled studies involving 97 patients were included in the analysis. In the meta-analysis, MSC treatments were superior to placebo treatments in good mRS (MD −0.95, 95% CI [−1.39, −0.52]) or BI (MD 21.36, 95% CI [9.96, 32.75]) scores, and MSC treatments were not superior to placebo treatments in the rate of improvement of the NIHSS scores (MD −1.81, 95% CI [−4.123, 0.494]). MSCs were associated with neither decreased mortality nor stroke recurrence (risk ratio 0.58 and 0.59, respectively; *p-*value = 0.51 and *p-*value = 0.533, respectively).

**Conclusion:**

For patients with acute and subacute ischemic stroke who are eligible for further damage to neural tissue, MSCs achieve high efficacy and acceptable safety.

**Systematic review registration:**

Prospero, unique ID: CRD42023457655.

## 1 Introduction

Stroke is the second-most common cause of death worldwide, accounting for 11.6% of fatalities in 2019. The most common type of stroke is ischemic stroke, accounting for 62.4% of all stroke incidents globally in 2019. Worldwide, 77.19 million people had an ischemic stroke in 2019, which resulted in 63.48 million disability-adjusted life years and 3.29 million fatalities (El-Hajj et al., 2016). The Middle East experiences a varying incidence of ischemic stroke, with reported rates ranging from 43.17 to 164 per 100,000 population per year (El-Hajj et al., 2016).

The standard therapy for acute ischemic stroke is tissue plasminogen activator therapy, which is a clot-busting medication that must be administered within 4.5 h of symptom onset (Broderick et al., 2013). Additionally, endovascular thrombectomy, a procedure that involves physically removing the clot from the blocked blood vessel, is also considered standard therapy for select patients with acute ischemic stroke (Badhiwala et al., 2015).

Recent research has suggested that stem cell therapy may hold promise as a potential treatment option for acute and subacute ischemic stroke. Stem cells can differentiate into various cell types and can potentially regenerate damaged tissue, making them a promising therapeutic approach for neurological disorders such as stroke (Ebrahimi et al., 2021).

Several types of stem cells can be used in the treatment of ischemic stroke, including embryonic stem cells, induced pluripotent stem cells, mesenchymal stem cells (MSCs), and neural stem cells (Marei et al., 2018). Embryonic stem cells are derived from embryos and can differentiate into any cell type in the body. These cells have the potential to replace damaged neurons and restore lost function in the brain after a stroke (Cui et al., 2010). MSCs are derived from the bone marrow or adipose tissue and can differentiate into several cell types, including neural cells. MSCs have been shown to have neuroprotective effects and can improve functional recovery after stroke (Li et al., 2016).

In this review, we sought to examine the effectiveness and safety of stem cell therapy for acute and subacute ischemic stroke. To evaluate the effect of stem cell therapy on functional outcomes, rates of mortality, and stroke recurrence in patients with acute and subacute ischemic stroke, we conducted a systematic literature search and analysis. Despite mixed findings, the potential advantages of stem cell treatments for acute and subacute ischemic stroke remain unknown. Therefore, it is crucial to properly evaluate the available research in order to present an up-to-date summary of the information on MSC therapy for acute and subacute ischemic stroke.

## 2 Methods

Throughout this systematic review and meta-analysis, we adhered to the Preferred Reporting Items for Systematic Reviews and Meta-Analysis (PRISMA statement) criteria (Page et al., 2021). The *Cochrane Handbook of Systematic Reviews and Meta-Analyses of Interventions*, version 5.1.0, was strictly followed in the execution of the techniques (Cumpston et al., 2019; Shaheen et al., 2023).

### 2.1 Eligibility criteria

We included all studies satisfying the following criteria:

Population: patients with acute and subacute ischemic stroke.
Intervention: MSCs (any dose).
Comparator: placebo.
Outcomes: We included studies reporting at least one of the following outcomes: (1) clinical improvement in National Institutes of Health Stroke Scale (NIHSS) scores, (2) good modified Rankin Scale (mRS) scores (0–1), (3) Barthel Index (BI) scores, (4) mortality, and (5) stroke recurrence.
Study design: Only randomized controlled trials (RCTs).

We excluded studies that were not in the English language and studies that used an observational design.

### 2.2 Literature search

We performed a comprehensive literature search of four electronic databases (PubMed, Scopus, Web of Science, and Cochrane CENTRAL) from inception until July 2023 using this search query ((Stem cells OR Stem Cell OR Progenitor Cell^*^ OR Mother Cell^*^ OR Colony-Forming Unit^*^) AND (Ischemic Stroke^*^ OR Cryptogenic Stroke^*^ OR Cryptogenic Embolism Stroke^*^ OR Wake-up Stroke^*^ OR wake up stroke^*^)). All duplicates were removed, and all references in the included articles were screened manually for any eligible studies.

### 2.3 Screening of the literature search results

The literature search results passed a two-step screening process. All paper titles and abstracts were initially reviewed for eligibility. The full-text articles of accepted abstracts were then obtained and checked for acceptance.

### 2.4 Data extraction

A standard data extraction sheet was used for data extraction. The retrieved data contained the following: (1) study characteristics, (2) study characteristics, (3) risk-of-bias domains, and (4) outcome measures.

### 2.5 Outcome measures

#### 2.5.1 mRS

The mRS is a scale with values ranging from 0 to 6 that is frequently used to measure the level of dependency or disability in people who have experienced a stroke or other neurological diseases (Runde, 2019). For statistical purposes, studies provide data as means and standard deviations.

#### 2.5.2 BI

The BI is a tool that is frequently utilized in rehabilitation settings. When someone enters a rehabilitation program, their functional state is evaluated, their progress is tracked over time, and the efficacy of rehabilitation therapies is assessed. A higher score on the BI, which has a total value that can vary from 0 to 100, indicates a higher level of independence (Prodinger et al., 2017). For statistical purposes, studies provide data as means and standard deviations.

#### 2.5.3 Clinical improvement in the NIHSS

The NIHSS is an established method for assessing the severity of neurological impairments caused by a stroke. These deficiencies, which include those in motor function, sensory perception, language skills, and vision, give unbiased information that aids medical personnel in assessing the success of interventions and the patient's condition during therapy. A higher score indicates more neurological impairments; the total score goes from 0 to 42 (National Cancer Institute, 2018). For statistical purposes, studies provide data as means and standard deviations.

#### 2.5.4 Mortality

Mortality is defined as the proportion of patients who died; it is represented as the risk ratio (RR) between the two groups.

#### 2.5.5 Stroke recurrence

The incidence of stroke recurrence is expressed as the RR between the two groups.

### 2.6 Synthesis of results

Using the metafor package in the R programming language, we created the generic inverse variance analysis and compared MSCs to placebos. Results for efficacy were presented as Mean Differences (MDs) with matching 95% confidence intervals.

### 2.7 Heterogeneity assessment

The chi-square test (Cochran's Q test) was used to assess the statistical heterogeneity between studies. The *I*^2^ was then calculated using the chi-square statistic, Cochran's Q, using the formula *I*^2^ = (Q – *df* ) ^*^ 100%/Q. Significant heterogeneity was defined as a chi-squared *p-*value of 0.1. *I*^2^ values of 0% indicated that there was no heterogeneity (Bergh, 2015).

### 2.8 Risk of bias across studies

Following the guidelines of the *Cochrane Handbook of Systematic Reviews of Interventions*, two authors independently evaluated the effectiveness of the included clinical trials. Using the quality assessment table from the same book (Cumpston et al., 2019), we looked at the likelihood of bias in a number of domains, including random sequence generation, allocation concealment, the blinding of participants and study staff, the blinding of outcome assessors, complete data, and selective outcome reporting.

### 2.9 Assessment of publication bias

Due to the low number of included RCTs (fewer than 10) and the advice given by Egger et al. (1997) we were unable to accurately quantify the publication bias using a funnel plot and Egger's test.

## 3 Results

### 3.1 Literature search results and study selection

**Figure 1:**
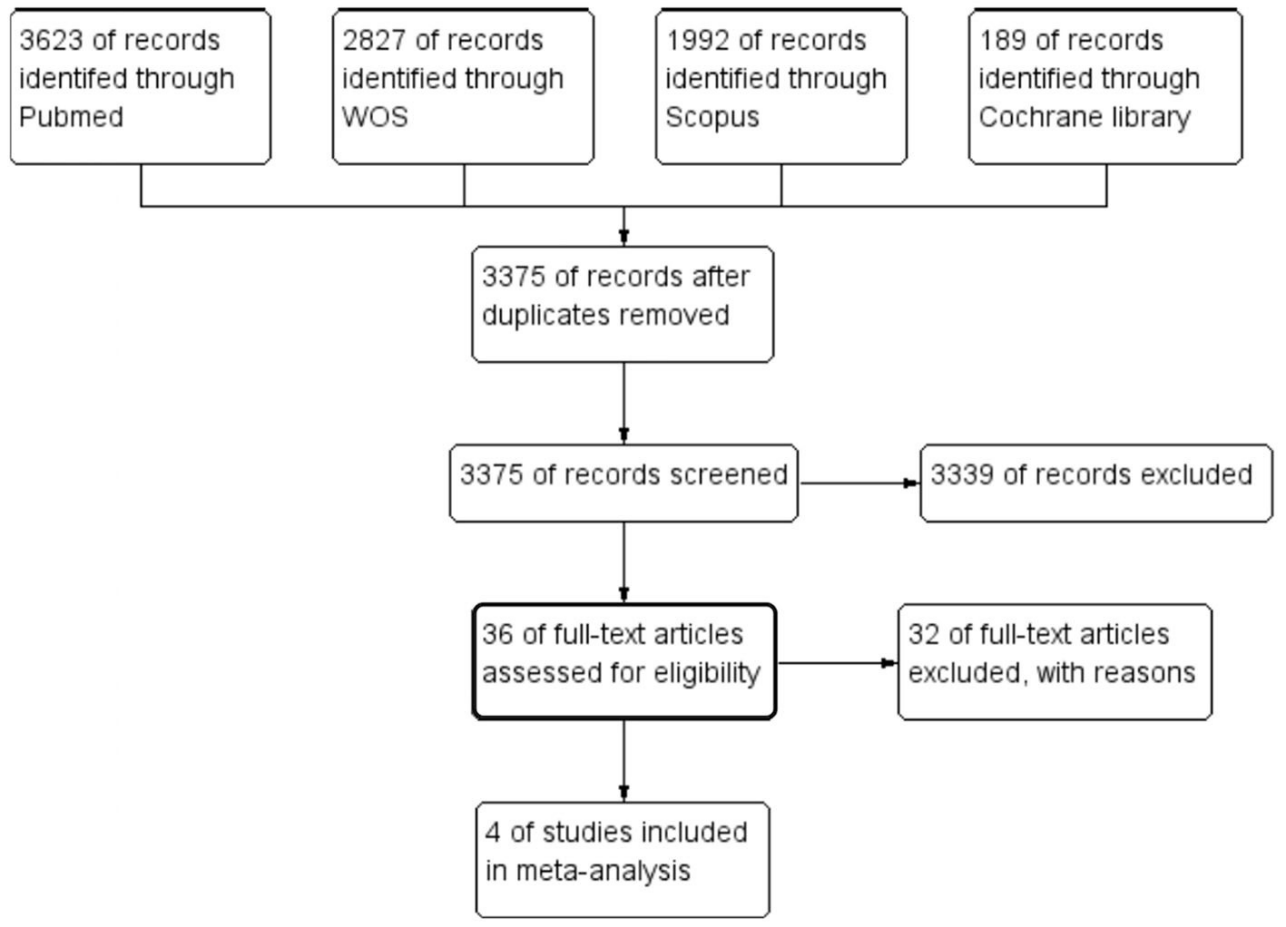
The preferred reporting items for systematic reviews and meta-analysis flow diagram of the study selection process.

Our search for relevant literature turned up 8,646 results. Of those, 36 articles qualified for full-text screening after being subjected to title and abstract screening. Four RCTs from these 36 trials were included in the meta-analysis. Additionally, no additional publications were included despite carefully searching the references of the listed research. The PRISMA flow diagram in Figure 1 provides the flowchart for the study selection approach.

### 3.2 Study characteristics

The four RCTs (Bang et al., 2005; Jaillard et al., 2020; Law et al., 2021; de Celis-Ruiz et al., 2022) involved 97 ischemic stroke patients. Table 1 provides more information on the characteristics of each study group, including age, sex distribution, baseline NIHSS scores, mRS scores, infarct volume, site of stroke, and follow-up durations. Table 2 summarizes the major information from various studies regarding the use of stem cells for treating stroke, the measured outcomes, and the key findings.

**Table 1:** Summary and characteristics of the population of included studies.

**Refernces**	**Study group**	**Age**	**Sex, male: n (%)**	**Baseline NIHSS**	**Baseline mRS**	**Infarct volume**	**Site of stroke**	**Follow up duration(s)**
Bang et al. (2005)	MSC group	63.0 ± 7.5	4 (80%)	10.6 ± 2.6	4.8 ± 5	127.4 ± 70.3	Middle cerebral artery (MCA)	3 m, 6 m, 12 m
	Control group	59.3 ± 11.5	14 (56%)	11.6 ± 4.9	4.6 ± 0.7	89.1 ± 77.4	Middle cerebral artery (MCA)	3 m, 6 m, 12 m
de Celis-Ruiz et al. (2022)	AD-MSC group	78 (70.5–82)	1 (11.1%)	10 ± 7.64	mRS 0 = 7 (77.8%) mRS 1 = 2 (22.2%)	43.22 (37.57–94.01)	Middle cerebral artery (MCA)	2 h, 24 h, 7 d, 3 m, 6 m, 12 m, 18 m, 24 m
	Control group	76 (69–80.25)	3 (30.0%)	8.83 ± 4.81	mRS 0 = 9 (90%) mRS 1 = 1 (10%)	88.165 (55.06–130.75)	Middle cerebral artery (MCA)	2 h, 24 h, 7 d, 3 m, 6 m, 12 m, 18 m, 24 m
Jaillard et al. (2020)	MSC group	55 (46–58)	11 (68.8%)	14 ± 6.5	3.83 ± 0.4	84 ± 66.65	Middle cerebral artery (MCA)	6 m, 12 m, 24 m
	Control group	53 (45–63)	11 (73.3%)	13 ± 4.09	3.74 ± 0.178	113 ± 48.15	Middle cerebral artery (MCA)	6 m, 12 m, 24 m
Law et al. (2021)	BM-MSC group	54.6 (13.2)	8 (88.89%)	17.166 ± 5.68	4.33 ± 0.874	68.2 ± 41	Middle cerebral artery (MCA)	1.5 m, 3 m, 6 m, 9 m, 12 m
	Control group	64 (13.9)	2 (25%)	15.5 ± 10.27	4.5 ± 0.89	38 ± 30.1	Middle cerebral artery (MCA)	1.5 m, 3 m, 6 m, 9 m, 12 m

**Table 2:** Summary and characteristics of the included studies.

**References**	**Population**	**Intervention**	**Types of stem cells**	**Dose of injection**	**Frequency of injection**	**Control group**	**Measured outcomes**	**Key findings**
Bang et al. (2005)	Acute Ischemic Stroke patients	IV infusion of MSC	Autologous Mesenchymal Stem Cell (MSC)	5 ^*^ 107	Twice	Standard medical care	NIHSS–BI–MRS–change in infarct sizze–ventricular dilation	Intravenous injection of *ex vivo*–cultured autologous MSCs is a safe and feasible method of treatment for ischemic stroke.
de Celis-Ruiz et al. (2022)	Acute Ischemic Stroke patients	Intravenous infusion of allogeneic AD-MSCs coupled with conventional treatment	Adipose tissue–derived mesenchymal stem cells (AD-MSCs)	1 million cells per kilogram	-	Conventional treatment for ischemic stroke according to the valid guidelines	NIHSS, infarct size, mRS, blood biomarkers.	The intravenous administration of AD-MSCs within the first 2 weeks of ischemic stroke onset is safe at 24 months of follow-up. Although no efficacy end points were statistically significant between treatment groups
(Jaillard et al., 2020)	Subacute Ischemic Stroke patients	Received IV injection of MSCs coupled with rehabilitation MSCs	Mesenchymal stem cells (MSCs)	The first ten patients assigned to treatment received low-dose MSCs (100 million) and the next ten patients received high-dose MSCs (300 million)	Once	Rehabilitation alone	mRS- BI- NIHSS- n-NIHSS–Motor FMS- MI-BA 4a -MI-BA 4p	Autologous MSC treatment is safe and feasible for treating moderate to severe stroke.
Law et al. (2021)	Subacute Ischemic Stroke patients	Standard medical care and culture expanded autologous BMMSCs	Autologous bone marrow derived MSCs (BMMSCs)	2.05 ± 0.20 ^*^ 106 BMMSCs per kg	Once	Standard medical care, which included treatment to prevent recurrence, optimal control of risk factors and post-stroke follow-up rehabilitative therapies.	NIHSS, mRS, BI, Infarct volume change	BMMSCs administered intravenously in the subacute period following MCA infarct were safe but did not improve functional outcome at 12 months. Improvements in radiological outcome were observed in the treatment group.

### 3.3 Risk of bias within studies

**Figure 2:**
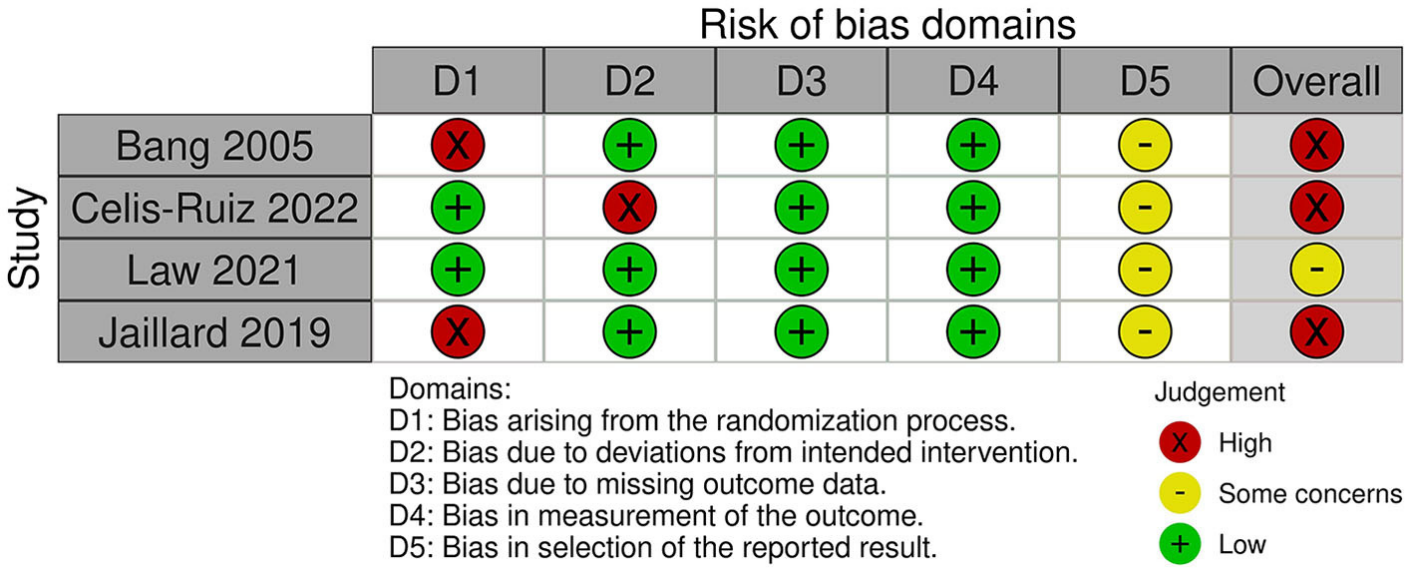
Risk of bias 2.

According to the Cochrane risk-of-bias assessment method, the included studies' quality ranged from moderate concerns to high risk. Due to the patients' and research staff's lack of blinding, two studies had a high risk of performance bias, and one study had a high risk of proper analysis. Except for the 2005 Bang trial, which did not disclose the methods used for sequence generation or allocation concealment, all studies had acceptable random sequence generation and minimal risk of selection bias in the allocation procedure. In 2019, Jaillard conducted an open-label RCT. Figure 2 provides the specific risk-of-bias domains by study ID (Sterne et al., 2019).

### 3.4 Clinical improvement in the NIHSS

**Figure 3:**
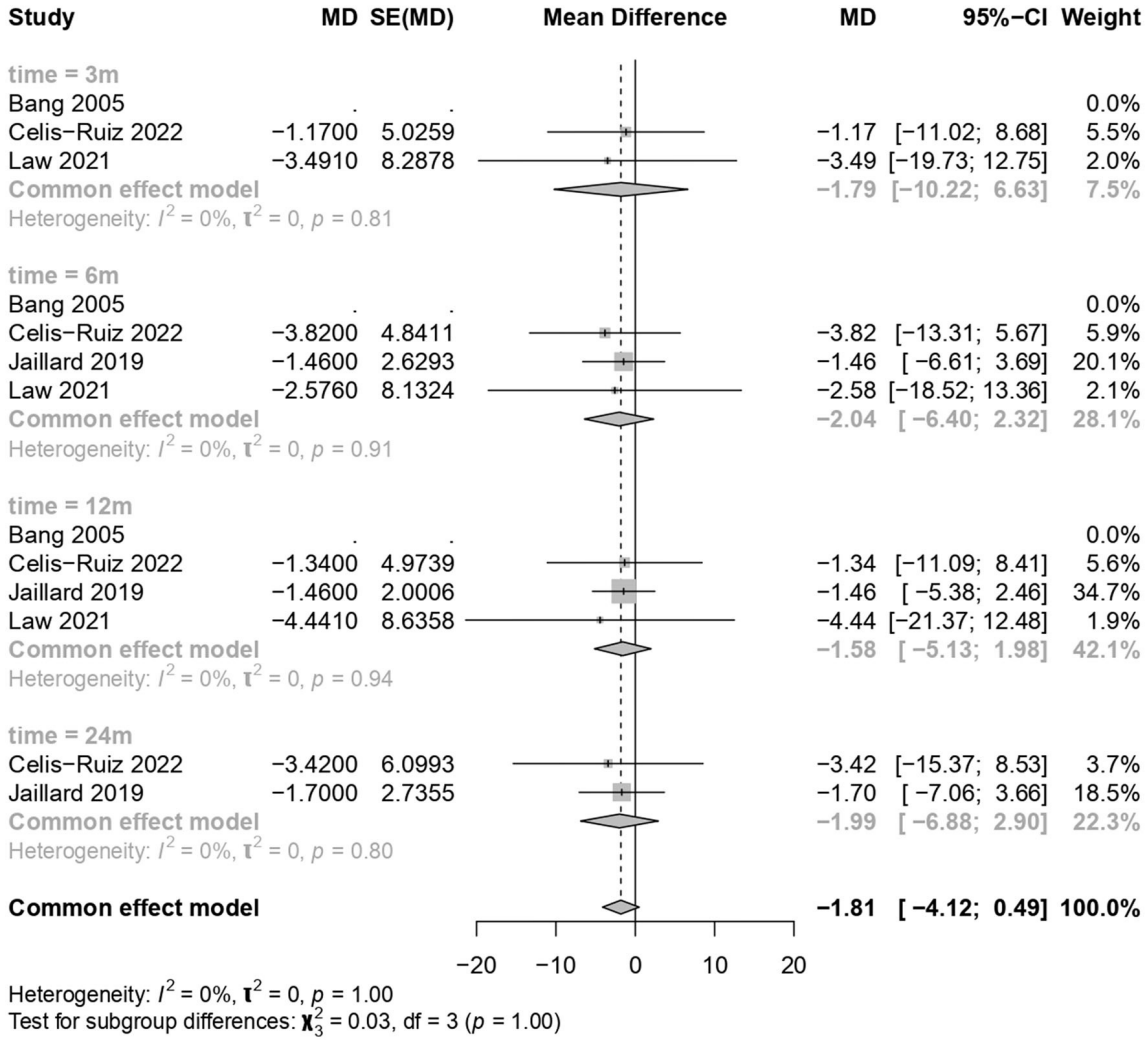
Forest plot for clinical improvement in the national institutes of health stroke scale.

MSC treatments showed no significant improvement compared to the placebo treatment (common effect model; MD −1.81, 95% CI [−4.123; 0.494], *p-*value = 0.1234; Figure 3). The calculated effect size using the inverted variance method was not statistically significant (*p* = 0.1234), indicating that the treatment strategies did not have a significant impact on the NIHSS scores in the analyzed studies. Pooled studies were homogenous (*I*^2^ = 0.0%; chi-square *p* = 1.00). Subgroup analyses were conducted based on different time points (3 months, 6 months, 12 months, and 24 months). A test for subgroup differences was performed to evaluate whether there were significant differences between the subgroups. The “between-groups” *p-*value was 1.00, indicating no significant subgroup differences. The “within-groups” *p-*value was 1.00, suggesting consistency within the subgroups.

### 3.5 Good neurological outcome on the mRS

**Figure 4:**
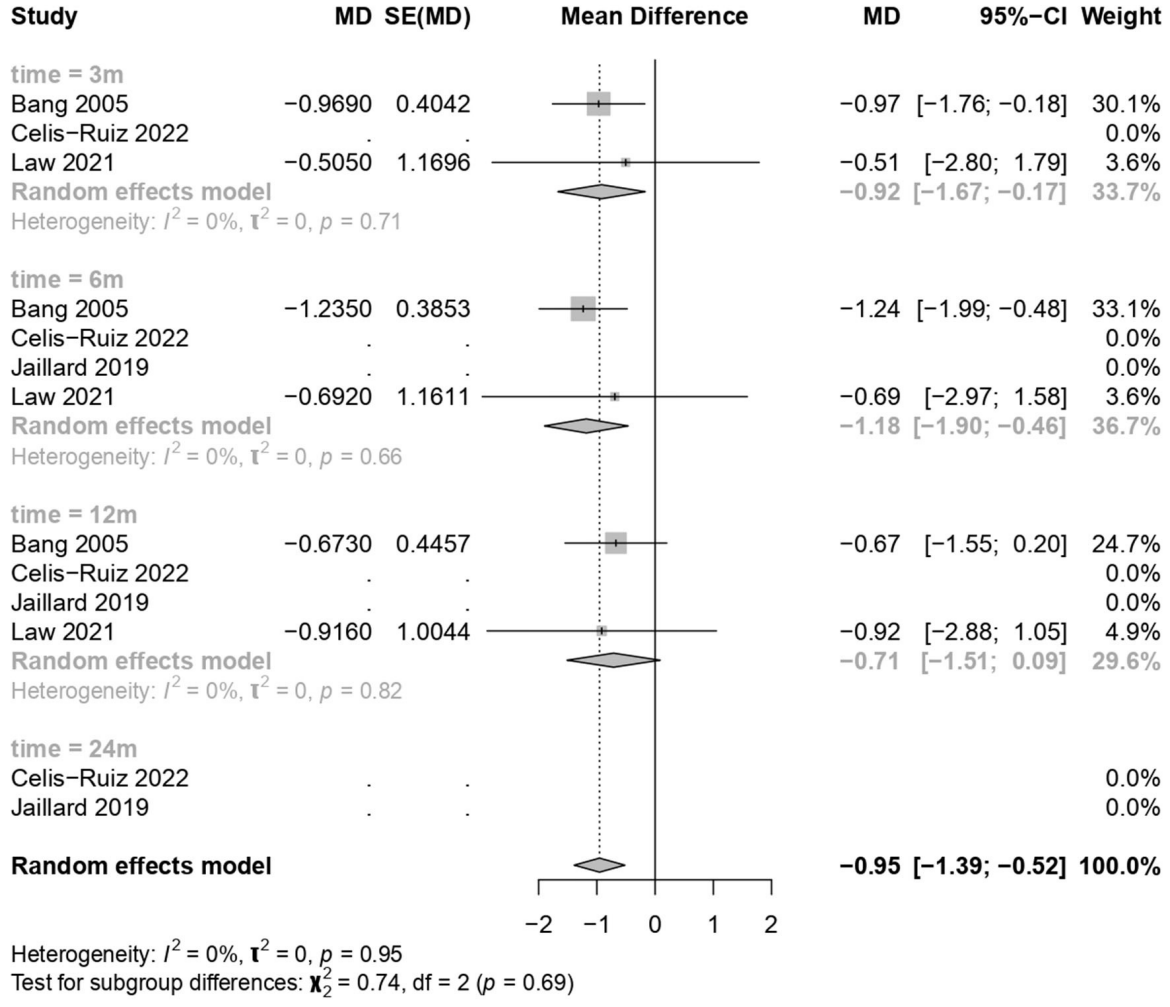
Forest plot for good neurological outcome on modified Rankin scale.

MSC treatments showed a significant improvement over the placebo treatment (common effect model; MD −0.95, 95% CI [−1.39; −0.52], *p-*value < 0.0001; Figure 4). The calculated effect size using the inverted variance method was statistically significant (*p* < 0.0001), indicating that the treatment strategies had a significant impact on the mRS scores in the analyzed studies. Pooled studies were homogeneous (*I*^2^ = 0.0%; chi-square *p* = 0.95). Subgroup analyses were conducted based on different time points (3 months, 6 months, and 12 months). A test for subgroup differences was performed to evaluate whether there were significant differences between the subgroups. The between-groups *p-*value was 0.6894, indicating no significant subgroup differences. The within-groups *p-*value was 0.9430, suggesting consistency within the subgroups.

### 3.6 Clinical improvement in the BI

**Figure 5:**
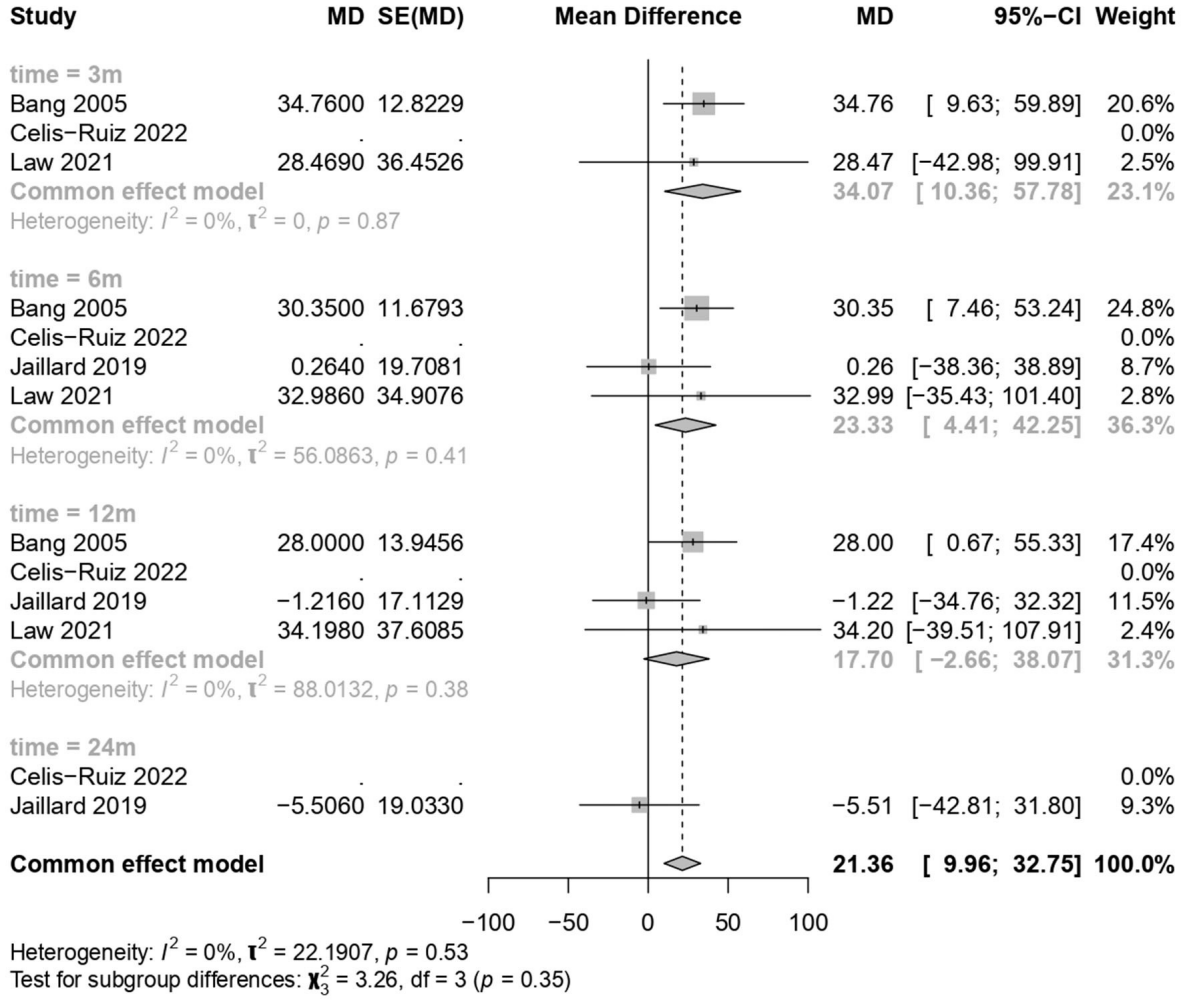
Forest plot for clinical improvement in the Barthel Index.

MSC treatments showed a significant improvement over the placebo treatment (common effect model; MD 21.36, 95% CI [9.96, 32.75], *p-*value = 0.0002; Figure 5). The calculated effect size using the inverted variance method was statistically significant (*p* = 0.0002), indicating that the treatment strategies had a significant impact on the BI scores in the analyzed studies. Pooled studies were homogeneous (*I*^2^ = 0.0%; chi-square *p* = 0.53). Subgroup analyses were conducted based on different time points (3 months, 6 months, 12 months, and 24 months). A test for subgroup differences was performed to evaluate whether there were significant differences between the subgroups. The between-groups *p-*value was 0.35, indicating no significant subgroup differences. The within-groups *p-*value was 0.57, suggesting consistency within subgroups.

### 3.7 Mortality rates

**Figure 6:**
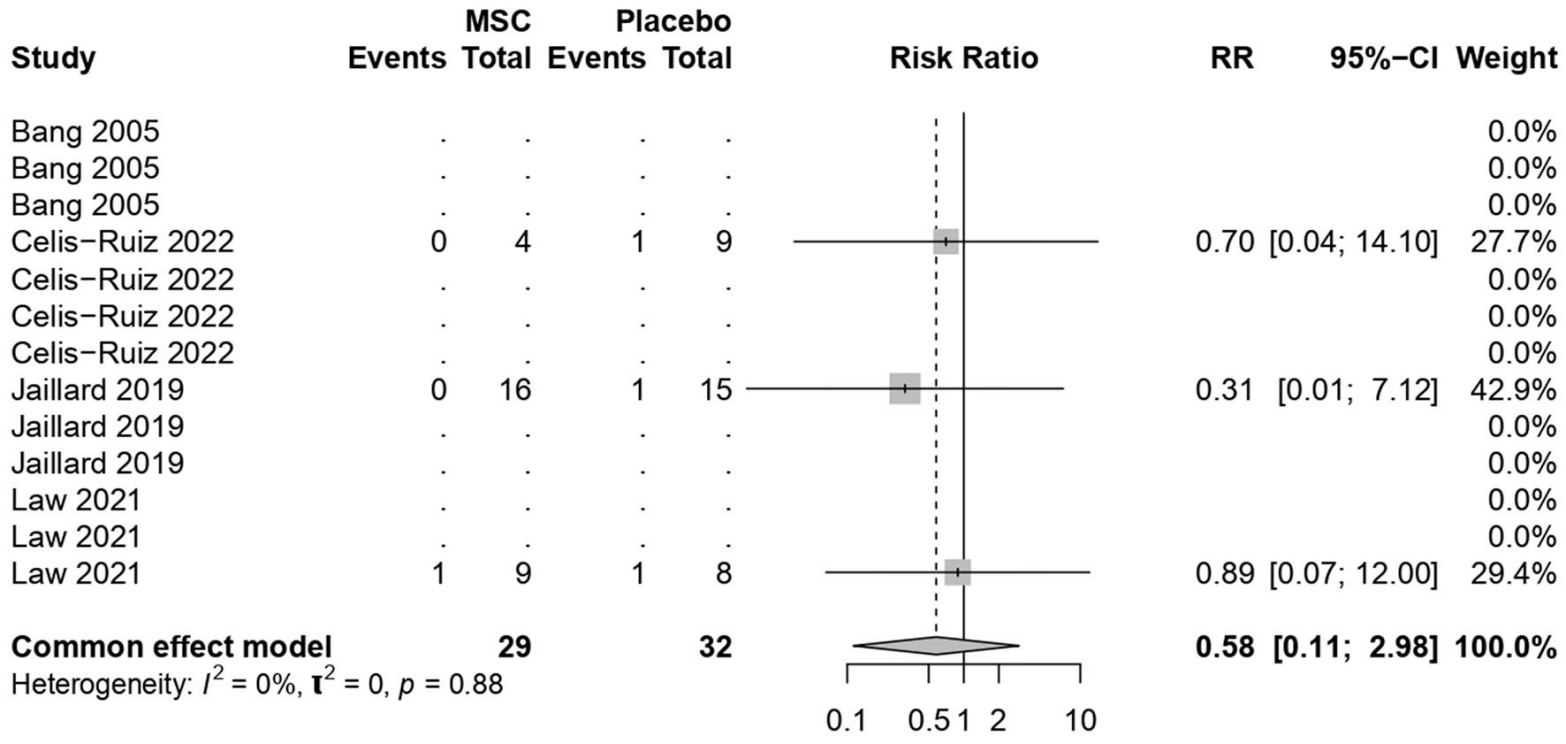
Forest plot for mortality rates.

MSC treatments were not associated with increased mortality (common effect model; RR 0.58, 95% CI [0.11, 2.97], *p-*value = 0.51; Figure 6). The calculated effect size using the Mantel–Haenszel method was not statistically significant (*p* = 0.51), indicating that the treatment strategies did not have a significant impact on the mortality rate in the analyzed studies. Pooled studies were homogeneous (*I*^2^ = 0.0%; chi-square *p* = 0.53).

### 3.8 Stroke recurrence rates

**Figure 7:**
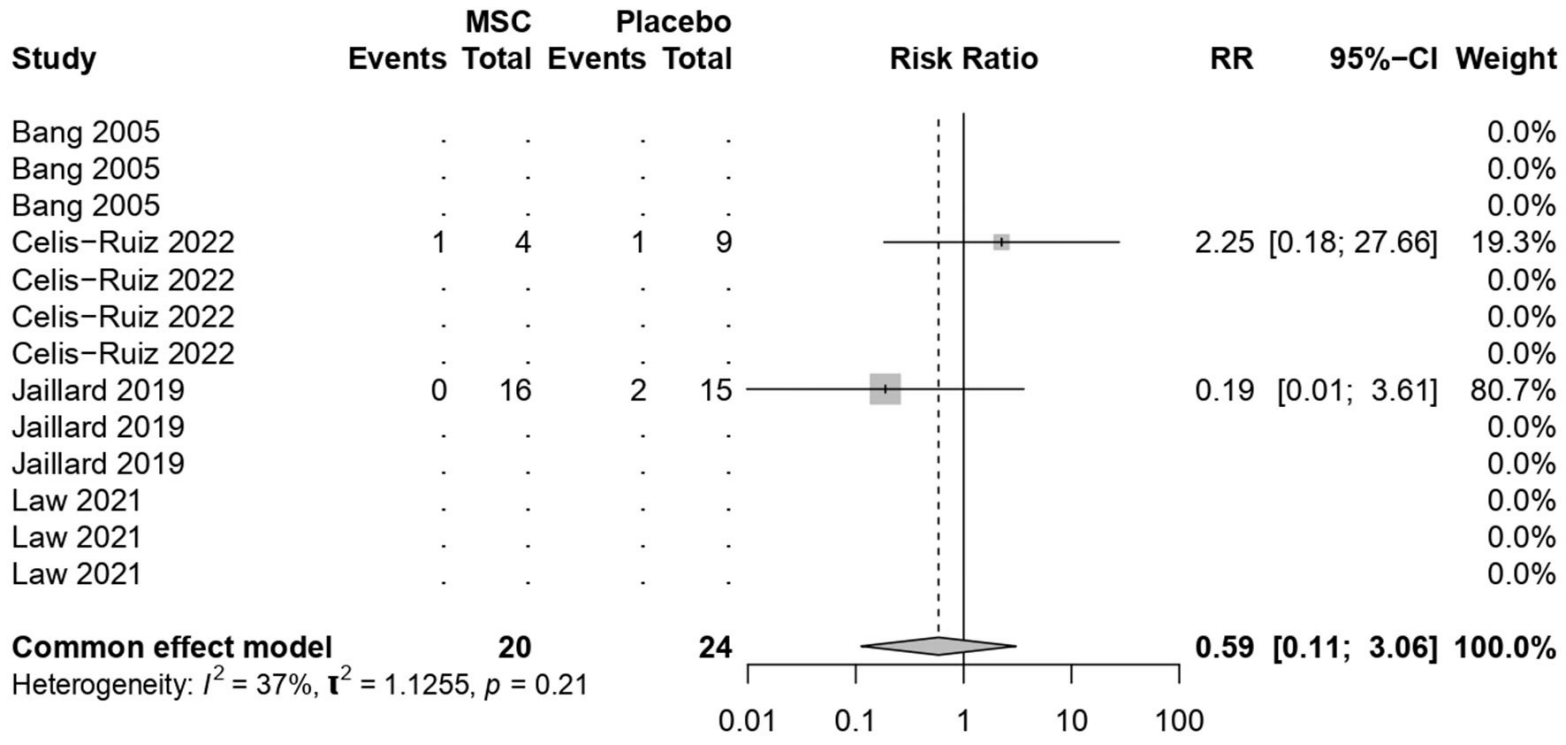
Forest plot for stroke recurrence rates.

MSC treatments were not associated with increased mortality (common effect model; RR 0.59, 95% CI [0.1122; 3.0588], *p-*value = 0.53; Figure 7). The calculated effect size using the Mantel–Haenszel method was not statistically significant (*p* = 0.53), indicating that the treatment strategies did not have a significant impact on the mortality rate in the analyzed studies. Pooled results were (*I*^2^ = 36.5%; chi-square *p* = 0.21). The moderate *I*^2^ value, along with the non-significant chi-square test, suggests that the observed heterogeneity might not be strong enough to undermine the overall findings of the stroke recurrence. However, potential sources of heterogeneity, such as sample size, among the included studies need further exploration and interpretation.

## 4 Discussion

### 4.1 Summary of the main findings

The meta-analysis findings from different outcome measures are as follows: (1) Clinical improvement in the NIHSS scores did not show significant differences between the MSCs and placebo groups (MD −1.81, 95% CI [−4.123, 0.494], *p* = 0.1234), indicating a negligible impact on the scores. (2) On the mRS outcomes, MSC treatments demonstrated a significant positive effect (MD −0.95, 95% CI [−1.39, −0.52], *p* < 0.0001), indicating a substantial improvement over placebo treatments. (3) The BI scores showed significant improvement with the use of MSCs (MD 21.36, 95% CI [9.96, 32.75], *p* = 0.0002). (4) The mortality and stroke recurrence rates did not exhibit significant differences between MSC and placebo treatments. (5) Heterogeneity was low for most analyses (*I*^2^ = 0.0%) except for stroke recurrence rates (*I*^2^ = 36.5%), where moderate heterogeneity was observed (chi-square *p* = 0.21). Despite this, the moderate *I*^2^ value, along with non-significant chi-square tests, suggests that heterogeneity might not significantly impact the overall findings. Furthermore, subgroup analyses and tests for subgroup differences showed consistent results within subgroups, reinforcing the consistency of the treatment effects across different time points.

### 4.2 Explanation of the study findings

Adult stem cells, known as MSCs, have the capacity to differentiate into a variety of mesoderm-derived cells, including osteoblasts, chondrocytes, myoblasts, and adipocytes, among others. They can be present in the bone marrow, fat tissue, and other connective tissues, among other bodily tissues (Orbay et al., 2012). MSCs are advantageous in the therapeutic setting due to their multipotency, immunomodulation, secretome, and low immunogenicity (Miceli et al., 2021). MSCs are being researched for a variety of therapeutic uses due to these attributes, including the treatment of neurological conditions including acute and subacute ischemic stroke (Li et al., 2016), as well as others like Parkinson's disease (Kitada and Dezawa, 2012) and Alzheimer's disease (Lee et al., 2012). MSCs are thought to provide neuroprotection, enhanced neurological recovery, and immune response regulation, according to a preclinical study (Uccelli et al., 2011). All of these elements benefit ischemic stroke patients by reducing the severity of tissue damage, preventing more cell death, and reducing oxidative stress, all of which are caused by the heightened immune response that occurs during the acute phase of a stroke. Since MSCs encourage the growth of new blood vessels (angiogenesis) (Velazquez, 2007), which increases blood flow and oxygen supply to brain areas, functional outcomes—whether motor or cognitive—are improved. Based on the previously stated principles, the results of MSCs on the mRS and the BI are predictable.

### 4.3 Significance of the work

Despite the promising results of MSCs in animal models of stroke, RCTs on MSCs have utilized small sample numbers, making the results less credible due to their effect size. By assessing the overall effect size of intravenous MSCs in patients with acute and subacute ischemic stroke, the current meta-analysis offers robust evidence. The study was the most rigorous meta-analysis to date addressing this research question, analyzing data from four studies involving a total of 97 participants. The study provided the most recent information on the safety and effectiveness of MSCs in acute and subacute ischemic stroke patients in an attempt to aid decision-making.

### 4.4 Agreement and disagreement with previous studies

Other delivery routes and cell types besides MSCs were taken into consideration in earlier individual patient data meta-analyses of RCTs and non-randomized trials. Furthermore, these meta-analyses did not separate patients with acute stroke and chronic ischemic stroke into separate groups. Additionally, in 2022, Yang et al. (2022) used a prior Bayesian model–based network meta-analysis that heavily relied on animal studies. Other meta-analyses performed by Ouyang et al. (2019) and Sarmah et al. (2018) had similar issues. The current study, in contrast, is founded on trustworthy human data and attempts to inform decision-making by presenting the most recent information on the safety and effectiveness of MSCs in patients with acute and subacute ischemic stroke. We found that the stem cell group was not superior to the placebo group in terms of NIHSS scores in contrast to the findings of Li et al. (2020) which found that participants in the stem cell group had significant outcomes in NIHSS score than the placebo group as reported in RCTs; however, in non-randomized studies, stem cell groups were not superior to placebo groups. We believe that our findings were achieved by including three randomized studies in the analysis (Jaillard et al., 2020; Law et al., 2021; de Celis-Ruiz et al., 2022) that were missing in the analysis by Li et al. (2020).

We conducted a systematic review and meta-analysis of clinical trials in acute and subacute ischemic stroke that compared safety outcomes (such as death and adverse effects) and efficacy outcomes (measured by scales such as the NIHSS, the mRS, or the BI) between the groups receiving stem cell–based therapies and control groups. The study adhered to the procedures outlined in the *Cochrane Handbook of Systematic Reviews for Interventions*, and it was presented using the PRISMA declaration, which is the standard format for systematic reviews and meta-analyses. The study maintains the accuracy and comprehensiveness of the findings by applying these strict processes and including only studies that provide detailed descriptions of their methodologies and analyses.

### 4.5 Strength points and limitations

This study has several strengths, which are as follows: (1) The study was conducted according to the methods explained in the *Cochrane Handbook of Systematic Reviews for Interventions* and reported according to the PRISMA statement (Cumpston et al., 2019; Page et al., 2021). This ensured that the study followed rigorous guidelines for conducting and reporting systematic reviews. (2) For the literature search and identification, the researchers searched multiple electronic databases to ensure a comprehensive literature search. They also examined all existing records on clinicaltrials.gov, including stopped, incomplete, and ongoing studies. This thorough approach increases the likelihood of capturing relevant studies and data. (3) Only studies whose data were published as full-text journal articles with a complete description of the methods and analysis were included in this study. This inclusion criterion helps ensure that the selected studies provide sufficient information for a comprehensive analysis. However, this study also has a few limitations: (1) The current evidence on the safety and efficacy of MSCs in acute and subacute ischemic stroke patients is limited by the small number of available RCTs. This suggests that more research is needed to draw definitive conclusions. (2) The findings of this study may not be generalizable to the entire population of acute and subacute ischemic stroke patients. It is important to consider individual patient characteristics and other factors when interpreting the results.

### 4.6 Ongoing studies and implications for clinical practice

We are aware of several ongoing clinical trials on MSCs (clinicaltrials.gov identifier: NCT05522569, NCT04097652, NCT04434768, NCT04093336, NCT03384433, and NCT03186456) for which the data are not yet available. In the future, as further data from these ongoing RCTs become available, this current meta-analysis will provide the most up-to-date information on the safety and efficacy of MSCs in acute and subacute ischemic stroke patients.

## 5 Conclusion

The use of intravenous MSCs in acute and subacute stroke patients showed positive results in terms of the mRS and the BI, indicating improved functional recovery and quality of life. However, while there were no substantial changes in NIHSS scores, mortality rates, or stroke recurrence, these outcomes emphasize MSCs' potential to enhance rehabilitation and highlight the need for further research to uncover the complex impacts of MSC therapy on stroke outcomes.

## Data availability statement

The original contributions presented in the study are included in the article/supplementary material, further inquiries can be directed to the corresponding author.

## Author contributions

MH: Data curation, Methodology, Supervision, Validation, Writing – original draft, Writing – review & editing. JF: Conceptualization, Data curation, Methodology, Writing – original draft. JS: Data curation, Methodology, Writing – original draft. EA: Data curation, Writing – original draft. MH: Conceptualization, Methodology, Validation, Visualization, Writing – original draft, Writing – review & editing. HG: Writing – review & editing. AN: Writing – original draft, Writing – review & editing.
